# Cytogenetic markers using single-sequence probes reveal chromosomal locations of tandemly repetitive genes in scleractinian coral *Acropora pruinosa*

**DOI:** 10.1038/s41598-021-90580-1

**Published:** 2021-05-31

**Authors:** Joshua Vacarizas, Takahiro Taguchi, Takuma Mezaki, Masatoshi Okumura, Rei Kawakami, Masumi Ito, Satoshi Kubota

**Affiliations:** 1 Kuroshio Science Program, Graduate School of Integrated Arts and Sciences, Kochi University, Nankoku, Kochi Japan; 2 Department of Nutrition, Faculty of Health Sciences, Kochi Gakuen University, Kochi, Kochi Japan; 3 Kuroshio Biological Research Foundation, Otsuki, Kochi Japan; 4 Sea Nature Museum Marine Jam, Kaiyo, Tokushima Japan; 5 Faculty of Agriculture and Marine Science, Kochi University, Nankoku, Kochi Japan

**Keywords:** Genetic mapping, Chromosomes

## Abstract

The short and similar sized chromosomes of *Acropora* pose a challenge for karyotyping. Conventional methods, such as staining of heterochromatic regions, provide unclear banding patterns that hamper identification of such chromosomes. In this study, we used short single-sequence probes from tandemly repetitive 5S ribosomal RNA (rRNA) and core histone coding sequences to identify specific chromosomes of *Acropora pruinosa*. Both the probes produced intense signals in fluorescence in situ hybridization, which distinguished chromosome pairs. The locus of the 5S rDNA probe was on chromosome 5, whereas that of core histone probe was on chromosome 8. The sequence of the 5S rDNA probe was composed largely of U1 and U2 spliceosomal small nuclear RNA (snRNA) genes and their interspacers, flanked by short sequences of the 5S rDNA. This is the first report of a tandemly repetitive linkage of snRNA and 5S rDNA sequences in Cnidaria. Based on the constructed tentative karyogram and whole genome hybridization, the longest chromosome pair (chromosome 1) was heteromorphic. The probes also hybridized effectively with chromosomes of other *Acropora* species and population, revealing an additional core histone gene locus*.* We demonstrated the applicability of short-sequence probes as chromosomal markers with potential for use across populations and species of *Acropora*.

## Introduction

Karyotyping is the process of pairing homologous chromosomes and arranging them in order of decreasing lengths. Karyotype, the systematic presentation of chromosomes, reveals the chromosome number, aneuploidy, ploidy variation, structural rearrangements, and the sexual form of an organism through the sex chromosomes. A karyotype, with its distinct markers, also provides the physical structure for cytogenetic and gene mapping. Aside from model organisms, karyotypes of most important crops and farmed animals are well documented, considering the important role of karyological data in genotyping and breeding^1,2^. However, karyotypes of other propagated animals, such as scleractinian corals, are poorly documented despite the increasing popularity of coral breeding as a strategy to rehabilitate degraded reefs^3–5^. Among 800 species of scleractinian corals, karyotypes of only 29 species have been reported, representing less than 4% of the total number of species^6^. For the karyotyped species, chromosome numbers are highly variable; for example in *Acropora*, the number ranges from 2n = 24 to 2n = 54^7^. This limited and varying karyological data for scleractinian corals can be attributed to the difficulty in constructing their karyotype due to their short (1–5 µm) and equally sized chromosomes^6,7^. Observations of unique banding patterns based on heterochromatic regions (Giemsa and C-bandings) were shown difficult for short chromosomes of some scleractinian corals^8,9^. These banding patterns and chromosomal lengths are features that are conventionally used in pairing homologous chromosomes to construct the karyotype. Karyotyping of corals has recently been improved with the use of fluorescence in situ hybridization (FISH), which provides a higher resolution that aids the observation of chromosomes by targeting gene loci as chromosomal markers^8–11^. This improvement revealed a chromosome number (2n) of 28 for most of the species of scleractinian corals and suggested slight variations in the number even within the species^9^. However, to gain a better understanding of these karyotypic variations, effective FISH probes that can be used across *Acropora* populations and species must be developed.

In cytogenetic analysis using FISH, large BAC probes (> 100 kbp) are commonly used because they target long regions of the chromosomes, creating bright and broad hybridization signals. However, due to the size of BAC probes, they may partly or largely contain simple tandem repeats (e.g., microsatellites), the lengths and composition of which vary between individuals and populations. This necessitates cross validation when applying BAC probes outside the tested individual^12^. In contrast, short probes that target only the conserved regions are potentially useful across populations and related taxa. However, to produce a bright FISH signal, the target gene needs to be either immensely long (> 6 kbp) or tandemly repeated. Fortunately, the nuclear ribosomal RNA (rRNA) genes and the core histone genes have highly conserved and repetitive properties, and their loci can therefore be detected using FISH employing only short probes containing the sequence of a single array that compose the tandem repeats. In contrast to large BAC probes, short probes (< 2 kbp) are also easier to develop with standard PCR and cloning procedures.

In this study, the loci of sequences associated to tandemly repetitive genes (5S rRNA and core histone genes) were detected in the chromosomes of *Acropora pruinosa* using suitable short single-sequence FISH probes. We propose that the loci detected using only short probes can produce bright hybridization signals that can be used as chromosomal markers for the identification of chromosome pairs. To identify the chromosome number on which the loci were observed, a tentative karyotype was constructed based on average chromosomal lengths. The developed FISH probes were then applied to the chromosomes of other population of *Acropora pruinosa* and species (*Acropora muricata*) to test the range of its applicability. These results reveal the potential of short single-sequence probes as tools for identification and pairing of homologous pairs within *Acropora*.

## Results

### Karyological features and whole genome hybridization

**Figure 1:**
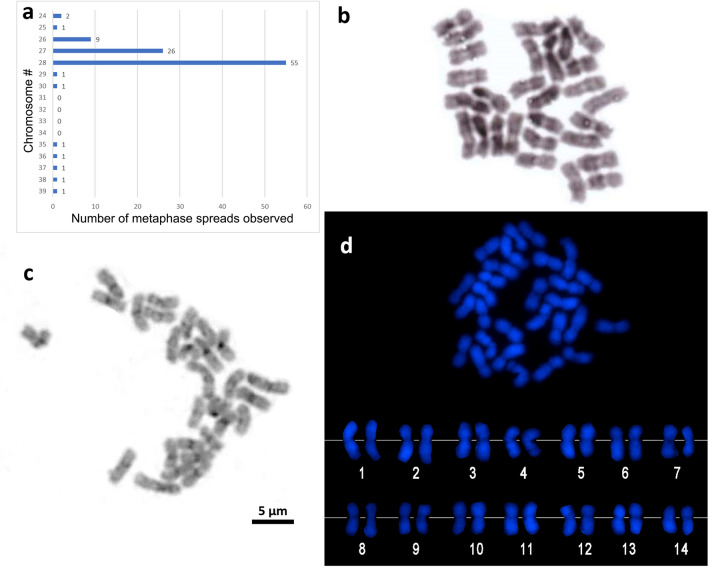
Chromosome numbers observed from 100 metaphase spreads (**a**). *Acropora pruinosa* chromosomes visualized by Giemsa staining (**b**) and C-banding (**c**). DAPI staining showing distinct centromeres (**d**).

The majority (55%) of the observed metaphase spreads (n = 100) of *A. pruinosa* had a chromosome number (2n) of 28 (Fig. 1a), followed by 27 (26%). Neither of the two conventional staining techniques (Giemsa and C-banding) provided a unique and clear banding pattern that could distinguish the homologous chromosomes (Fig. 1b,c). In C-banding, not all chromosomes showed a darkly stained centromeric region (Fig. 1c). On the contrary, 4',6-diamidino-2-phenylindole (DAPI) staining revealed constricted regions of the centromeres (Fig. 1d). Using the DAPI-stained chromosomes, their average centromere locations and individual lengths were measured, and chromosomes were arranged in order of decreasing lengths (Fig. 1d, Table 1). The centromere indices (0.54–0.57) indicated a centromeric characteristic for all the chromosomes (Table 1). Differences in chromosome lengths were not readily noticeable, in which the shortest chromosome was more than half (64.71% ± 4.3%) the size of the longest chromosome. To determine a heteromorphic pair, the size difference between each putative homologous chromosome was statistically compared (Supplementary Table S1). The size difference of the first homologous pair (chromosome 1) was found to be significantly larger than that of the other homologs (Table 1). This indicates that the first chromosome pair is heteromorphic in *A. pruinosa*.

**Table 1:** Morphometric characteristics of the chromosomes of *Acropora pruinosa* (n = 20 metaphase spreads).

Rank according to length	Long arm length (µm)	Chromosome length (µm)	Centromere index (long arm/total length)	Relative size (%)	Assigned chromosome #	Size difference between homologs (µm)*
1	1.89 ± 0.38	3.37 ± 0.67	0.56 ± 0.04	100	1	0.17 ± 0.11^a^
2	1.82 ± 0.37	3.2 ± 0.58	0.57 ± 0.04	95.18 ± 2.53		
3	1.77 ± 0.33	3.11 ± 0.57	0.57 ± 0.04	92.41 ± 3.18	2	0.08 ± 0.07^b^
4	1.69 ± 0.31	3.03 ± 0.56	0.56 ± 0.04	90.13 ± 3.66		
5	1.7 ± 0.32	2.98 ± 0.56	0.57 ± 0.03	88.45 ± 3.16	3	0.06 ± 0.07^bc^
6	1.62 ± 0.36	2.92 ± 0.54	0.55 ± 0.04	86.73 ± 3.43		
7	1.59 ± 0.29	2.87 ± 0.51	0.55 ± 0.04	85.48 ± 3.73	4	0.04 ± 0.03^bc^
8	1.6 ± 0.34	2.83 ± 0.50	0.56 ± 0.04	84.3 ± 3.4		
9	1.56 ± 0.28	2.8 ± 0.50	0.56 ± 0.04	83.19 ± 3.39	5	0.03 ± 0.05^bc^
10	1.55 ± 0.29	2.76 ± 0.48	0.56 ± 0.03	82.22 ± 3.9		
11	1.51 ± 0.30	2.73 ± 0.47	0.55 ± 0.04	81.33 ± 3.94	6	0.03 ± 0.02^bc^
12	1.52 ± 0.31	2.71 ± 0.48	0.56 ± 0.04	80.56 ± 4.08		
13	1.49 ± 0.28	2.67 ± 0.47	0.56 ± 0.04	79.52 ± 4.1	7	0.03 ± 0.04^bc^
14	1.43 ± 0.24	2.64 ± 0.46	0.54 ± 0.03	78.63 ± 3.91		
15	1.45 ± 0.31	2.61 ± 0.46	0.55 ± 0.04	77.82 ± 3.77	8	0.02 ± 0.02^bc^
16	1.41 ± 0.33	2.59 ± 0.46	0.54 ± 0.03	77.15 ± 3.93		
17	1.38 ± 0.27	2.56 ± 0.45	0.54 ± 0.03	76.13 ± 3.47	9	0.01 ± 0.01^c^
18	1.41 ± 0.25	2.54 ± 0.44	0.55 ± 0.03	75.71 ± 3.5		
19	1.41 ± 0.26	2.52 ± 0.45	0.56 ± 0.04	75.06 ± 3.46	10	0.03 ± 0.02^bc^
20	1.34 ± 0.23	2.5 ± 0.44	0.54 ± 0.03	74.31 ± 3.46		
21	1.35 ± 0.27	2.47 ± 0.44	0.55 ± 0.04	73.42 ± 3.25	11	0.02 ± 0.03^bc^
22	1.33 ± 0.25	2.44 ± 0.44	0.55 ± 0.04	72.72 ± 3.49		
23	1.3 ± 0.23	2.41 ± 0.43	0.54 ± 0.03	71.81 ± 3.28	12	0.04 ± 0.03^bc^
24	1.33 ± 0.29	2.38 ± 0.42	0.56 ± 0.04	70.8 ± 3.49		
25	1.3 ± 0.22	2.34 ± 0.42	0.56 ± 0.05	69.71 ± 3.27	13	0.06 ± 0.06^bc^
26	1.27 ± 0.28	2.29 ± 0.43	0.55 ± 0.03	67.97 ± 3.7		
27	1.23 ± 0.25	2.24 ± 0.41	0.55 ± 0.03	66.56 ± 3.87	14	0.06 ± 0.06^bc^
28	1.17 ± 0.25	2.18 ± 0.42	0.54 ± 0.03	64.71 ± 4.3		

*Different letters indicate significant differences (*P* < 0.05). Details of the analysis are shown in Supplementary Table S1.

**Figure 2:**
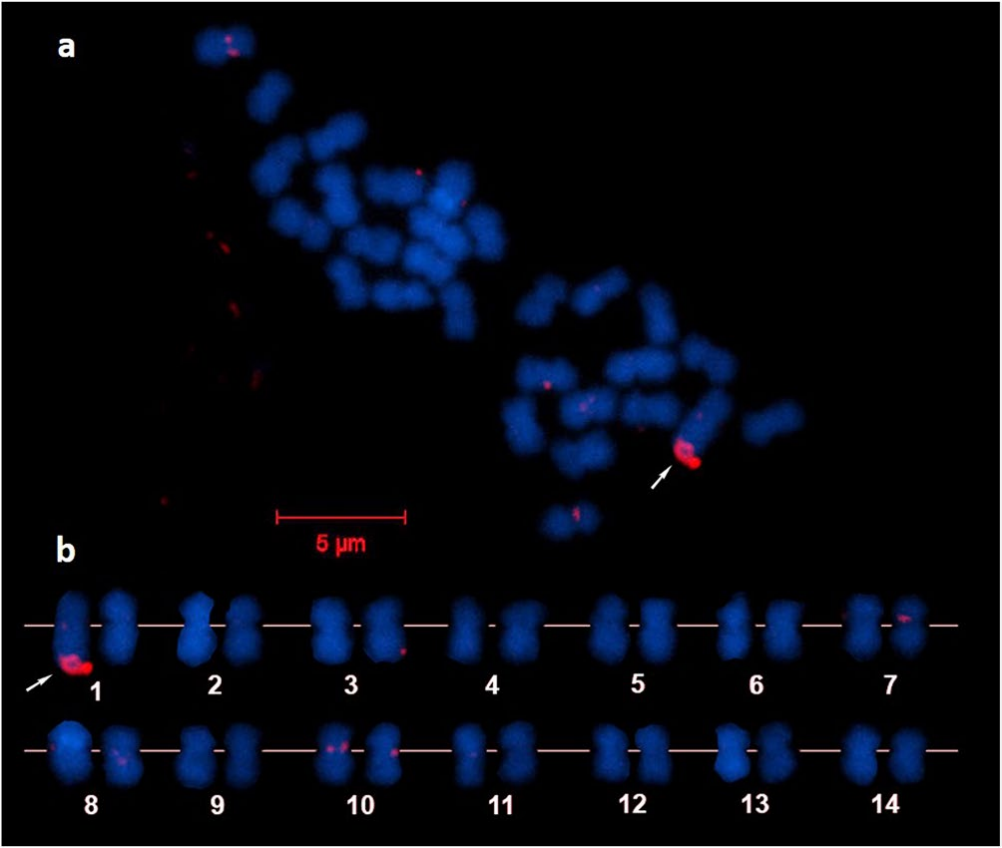
Whole genome hybridization of sperm DNA on chromosomes of *Acropora pruinosa* (**a**). Chromosomes arranged in order of decreasing length (**b**).

To assess the locations of all repetitive loci that are readily detected by FISH, whole genome hybridization (WGH) was conducted using a probe prepared from the whole genome of *A. pruinosa* sperm. Results showed several faint hybridization signals on some chromosomes, but a broad and intense signal was detected at the telomeric region of the q-arm of a single chromosome (Fig. 2a). The arrangement of chromosomes according to size revealed that the intense hybridization signal was on the longer chromosome of the heteromorphic chromosome 1 (Fig. 2b). This indicates that a long and unique array of sequences was present only on this single chromosome and was absent from other chromosomes, as well as on its homologous pair. Because this hybridization pattern was observed on all metaphase spreads and across different embryos, we eliminated the possibility of allelic variation between the heteromorphic pair of chromosome 1. In addition, the location of the hybridization signal is the portion of the chromosome that is missing in its homologous pair (Fig. 2b), thus suggesting a region that may not have the function and characteristics of a locus.

### Probe hybridization and sequence characterization

**Figure 3:**
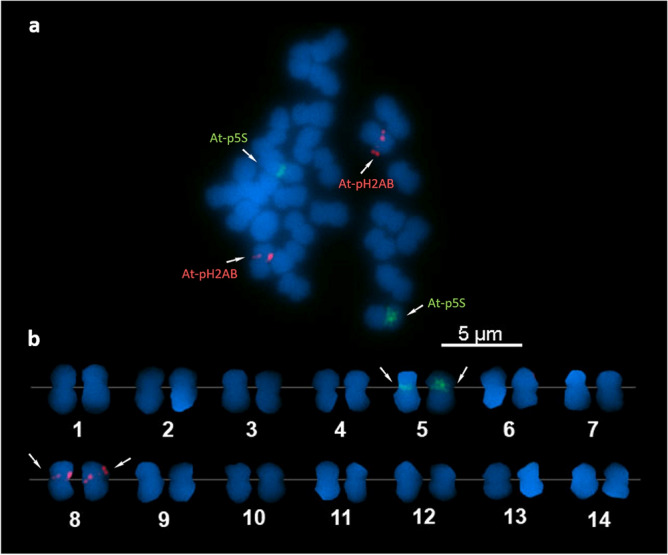
Fluorescence in situ hybridization image showing hybridization signals of the At-p5S probe labeled with digoxigenin-dUTP (green) and At-pH2AB probe labeled with Cy3-dUTP (red) in *Acropora pruinosa* chromosomes (**a**). Karyogram based on decreasing chromosome lengths (**b**).

Hybridization of the At-p5S and At-pH2AB probes revealed readily detected single loci in separate homologous pairs (Fig. 3). The hybridization with At-p5S and At-pH2AB probes manifested as band-like and dot-like signals, respectively. This indicates that the location of At-pH2AB is clustered but may include a relatively long interspersed region between arrays, whereas that of At-p5S is broader and more contiguous. Based on the average relative sizes of the chromosomes where the hybridization signals were detected, the At-p5S loci were located on chromosome 5 and the At-pH2AB loci were on chromosome 8 (Table 2).

**Table 2:** Characterization of the fluorescence in situ hybridization probes and their hybridization signals on the chromosomes of *Acropora pruinosa*.

FISH probe	Length	Sequence (GenBank accession)	Loci position in the chromosome	Fluorescence signal length (µm)	Relative length of chromosomal location (%)	Assigned chromosome number
At-WGH	–	–	Telomeric region of the q arm (one chromosome only)	0.62 ± 0.17	98.27 ± 5.05	1*
At-p5S	1731 bp	LC557013.1	p arm, near the centromere	0.40 ± 0.10	83.12 ± 6.03	5
At-pH2AB	813 bp	LC557014.1	p arm, near the centromere	Dot signal	77.42 ± 5.92	8

*Only the longer chromosome of the homologous pair.

**Figure 4:**
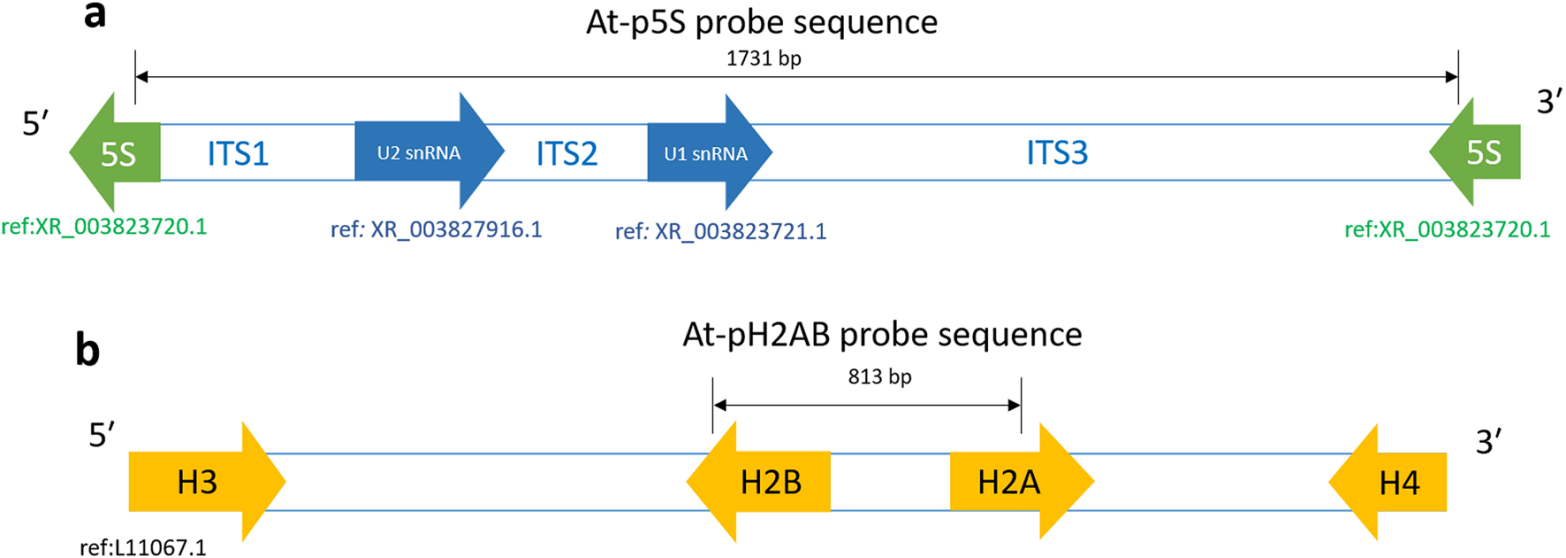
Characterization of the At-p5S (**a**) and At-pH2AB (b) probe sequences based on sequence alignment with their most homologous sequences from the GenBank.

Characterization of the probe sequence revealed that At-p5S is composed of small nuclear spliceosomal RNA genes (U1 and U2 snRNAs) and contains three interspacer regions (Fig. 4a). These regions were flanked by short sequences of the 5S rDNA, arranged in a head-to-tail fashion. The At-pH2AB probe is composed of two histone domains (H2A and H2B), separated by a spacer region (Fig. 4b). The two genes are arranged in a tail-to-tail fashion, which is typical among invertebrates^13–15^.

To confirm whether the short 5S rDNA sequence of the At-p5S probe was involved in the hybridization, we blasted the probe sequence (divided into identified regions) against the whole genome of *Acropora digitifera* (supplementary Table S2). Result of the analysis showed that the entire probe’s length including the short 5S rDNA sequences on both ends was present and tandemly repeated. The arrangement of 5S-ITS1-U2-ITS2-U1-ITS3-5S was also highly consistent within estimated length of 423,641 bp (supplementary Table S2, highlighted in yellow).

**Figure 5:**
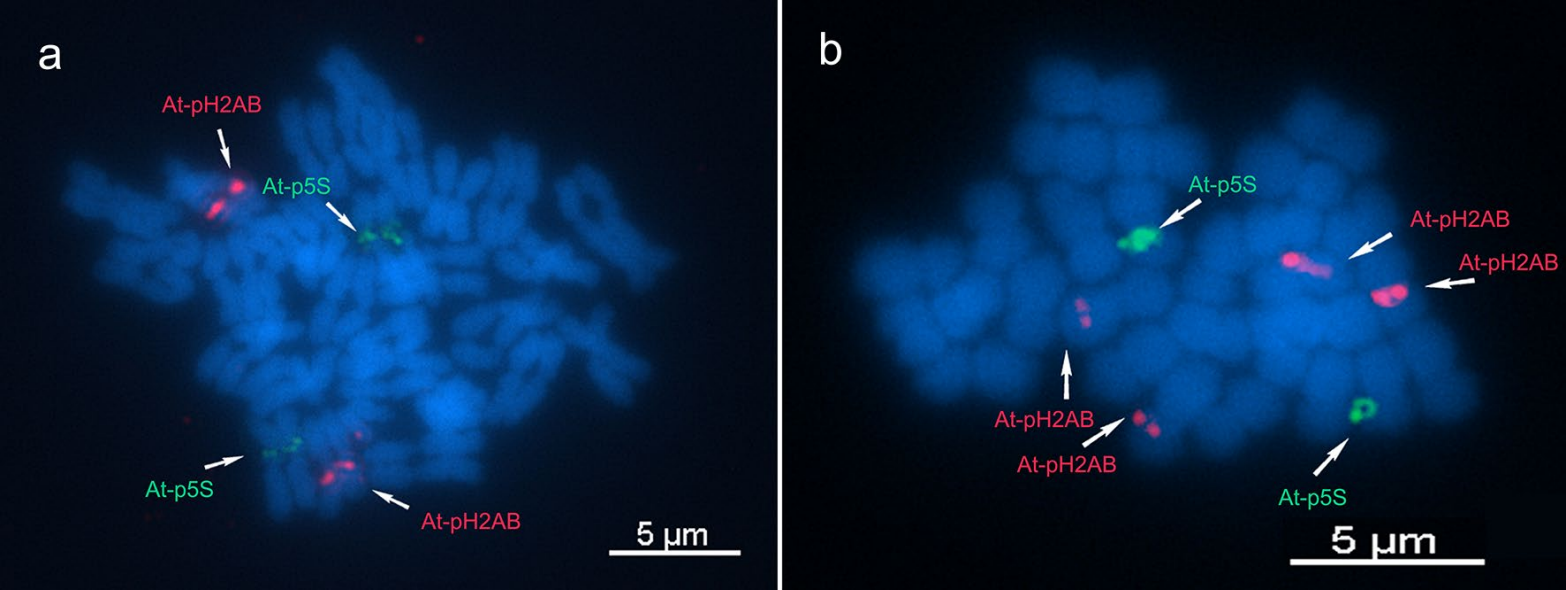
Fluorescence in situ hybridization image showing hybridization signals of the At-p5S (green) and At-pH2AB (red) probes on the chromosomes of *Acropora muricata* (**a**) and *Acropora pruinosa* Kochi (**b**).

The probes prepared from *A. pruinosa* were tested for the chromosomes of *A. muricata* and *A. pruinosa* Kochi. Hybridization signals were effectively detected in these two *Acropora* chromosomes (Fig. 5). In *A. muricata*, the hybridization pattern was the same as observed in *A. pruinosa* (one homologous pair for each probe). In addition, the loci were also observed at roughly the same chromosomal position, near the centromere of the p-arm (Fig. 5a). Conversely, in *A. pruinosa* Kochi*,* the hybridization signal for At-pH2AB was detected on two homologous pairs, with additional signal that was less intense than the other (Fig. 5b). This indicates that this locus contains fewer copies of core histone gene repeats than the other. Aside from the differences in signal intensity, the chromosomal positions of the additional At-pH2AB loci slightly departed from the centromere compared with those for the other At-pH2AB loci.

## Discussion

The chromosome number (2n = 28) of *A. pruinosa* agrees with those of other 18 species of *Acropora* and five other species from other coral genera (*Montipora* and *Fungia*)^7^. It is unclear whether the chromosome number 2n = 27 observed in this study was a result of missing one chromosome during mitotic preparations or it is another karyological characteristics in this coral species. Having two chromosome numbers (karyotypic mosaicism) is not uncommon in *Acropora*^7,9^. *Acropora pruinosa* Kochi was reported with chromosome numbers, 2n = 28 and 2n = 29, which was confirmed by the presence of an additional and unpaired chromosome in the case of 2n = 29^9^.

Large-scale hybridization signals on a single chromosome were observed using WGH in this study on *A. pruinosa* (2n = 28) as well as in a previous study on *A. pruinosa* Kochi (2n = 29)^9^. However, for *A. pruinosa* with even number of chromosomes, the presence of a unique chromosome with no apparent pair based on length and hybridization pattern might indicate the presence of heteromorphic pairs. In most animals, these heteromorphic pairs are often associated with sex chromosomes. Although the sex-linked loci and genes have been identified in the gonochoric coral *Corallium rubrum*^16^, the role of heteromorphic chromosomes in the sexual characteristics of scleractinians has not been explored. This investigation is particularly important in *Acropora* because colonies of some coral species may contain male or female polyps, aside from the well-known co-sexual polyps^17^. The heteromorphic pairs observed in this study were present in all mitotic cells, and we propose two mechanisms how these cells maintained to carry this unusually long chromosome: (1) After meiotic segregation in the hermaphroditic gonads, either the eggs or the sperms exclusively receive this chromosome, (2) a cycle that involves translocation of the portion of chromosome from the autosomes, causing the chromosome that receives it the longest one. The second mechanism has been demonstrated in other organisms, which involves translocation of the nucleolar organizer region (NOR) containing repetitive tandem arrays of 18S and 28S rRNA genes from autosomes to the telomeric end of sex chromosomes^18–20^. This NOR in the sex chromosomes functions in the pairing of X–Y chromosomes during meiosis^21^. This is also supported by the presence of 18/28S rDNA loci at the telomere of one of the longest chromosome pairs in *A. pruinosa* Kochi^9^. Further work must be conducted to characterize the sequence arrays that constitute this hybridization signal on the longest chromosome and to confirm whether this chromosome is associated with functioning as a sex chromosome.

The loci of the U1/U2 snRNA and core histone gene clusters showed intense hybridization signals on separate chromosome pairs. However, because the minimum sequence length of hybridization that can be readily detected in FISH is 6 kbp^15^, which is greater than the length of our probes (Table 2), it is possible that other loci composed of fewer or shorter arrays of the target genes exist. This is supported by the results of the experiment on the presence of several rDNA arrays obtained from subcloning, with shorter size of the target gene (LC557012, LC557015) that showed no hybridization signal. A sequence of similar length, but composed of indels (LC557016), compared with the identified repetitive histone array also showed no hybridization in FISH. Because these sequences were confirmed in the genome of *A. pruinosa*, we speculate that these arrays were either not repetitive (single-copy locus) or were short enough to be detected by FISH. Nonetheless, this study confirms the existence and chromosomal locations of highly clustered arrays of these genes. Studies have reported that this clustering of highly conserved genes is related to pseudogenes, which are acquired through hybridization of ancestral genes and have lost their coding potential^22,23^. Pseudogenes are implicated in the diversity of the nuclear ribosomal genes in *Acropora*, but only one rDNA sequence has been implicated to present across several species that are associated with pseudogenes^24^. It has also been reported previously that large clusters of pseudogenes consist of tRNAs and snRNAs on mammalian chromosomes^25,26^. Other identified pseudogenes that have repetitive gene copies in humans are the ribosome biogenesis protein gene (RLP24) and E3 ubiquitin-protein ligase gene (MDM2)^27^. Clustering of pseudogenes was also implicated in a mechanism to disable its function as a result of acquired mutations^28,29^. The arrangement of these genes in these clusters is tandemly repeated and lacks introns, and thus presumably arose from reverse transcription of mRNA, followed by multiple integration to specific regions in the chromosome^29–31^.

The linkage of snRNA genes and 5S rDNA sequence and their tandemly repetitive characteristics observed in this study was first reported for mollusks^32^. The same linkage involving U1, U2, and U5 snRNA genes was also found in fish^33^ and crustaceans involving only U1 snRNA^34^. Here, we report for the first time a tandemly repetitive linkage of 5S rDNA sequence and snRNA genes in the phylum Cnidaria. Although many FISH studies of single or multiple loci of repetitive 5S rRNA genes^35–37^ and snRNA genes^38,39^ have been reported, it is uncertain whether the loci observed in these studies may involve linkage to one another or to any other gene. We showed that repetitive linkage of snRNA and putative 5S rRNA genes produced a single locus on the chromosomes. Conversely, in fish, the loci of these two repetitive genes were not linked and were located on different chromosomes^40^.

Only the H2A and H2B genes arranged in a typical manner were confirmed to constitute the observed loci. However, in cnidarians, various arrangements of repetitive core histone genes, including H1, H3, and H4, have been documented^12^. In *Mytilus edulis*, aside from the core histone genes, the sequence of the solitary linker H1 gene is also tandemly repeated^41,42^. The loci of these solitary H1 gene clusters were found to be located on chromosome pairs different from core histone genes^43^. This suggests the possible presence of other repetitive histone loci that can be observed in scleractinian chromosomes. Surprisingly, a unique arrangement of repetitive arrays involving linkage between histone and 5S rRNA genes was observed among crustaceans^44^ and fish^45,46^.

The varying hybridization patterns of core histone probes in other *Acropora* population might suggest chromosomal rearrangements during the evolutionary processes within *Acropora*. In the genus *Mus*, locations of clusters of conserved genes are shifted across different chromosomes, providing evidence of genome reshuffling that occurred during its evolution^47^. Variations in the number of histone loci within closely related taxonomic groups have also been observed in other taxa. In bivalves, loci of histone genes are in two chromosome pairs in the mussel, *Mytilus galloprovinciali*^48^, and in the scallop, *Patinopecten yessoensis*^49^, but there is only one locus in the mussel species, *Perumytilus purpuratus*^50^ and in three other species of scallops (*Argopecten irradians*, *Chlamys farreri*, and *C. nobilis*)^49^.

We demonstrated that single-sequence probes containing conserved genes produced a readily detectable hybridization signal on the chromosomes of *A. pruinosa*. These probes also hybridized on chromosomes of other *Acropora* population and species and thus have a potential for use as chromosomal markers within the taxa. In addition, the single-sequence probes revealed the presence of other loci in other species, which revealed the differences in chromosome organization. This study may provide a foundation for discovering the loci of other tandemly repetitive genes, such as 18 and 28S rDNA that can be used as additional chromosomal markers for improved karyotyping of *Acropora*.

## Methods

### Sample collection and chromosome preparation

**Figure 6:**
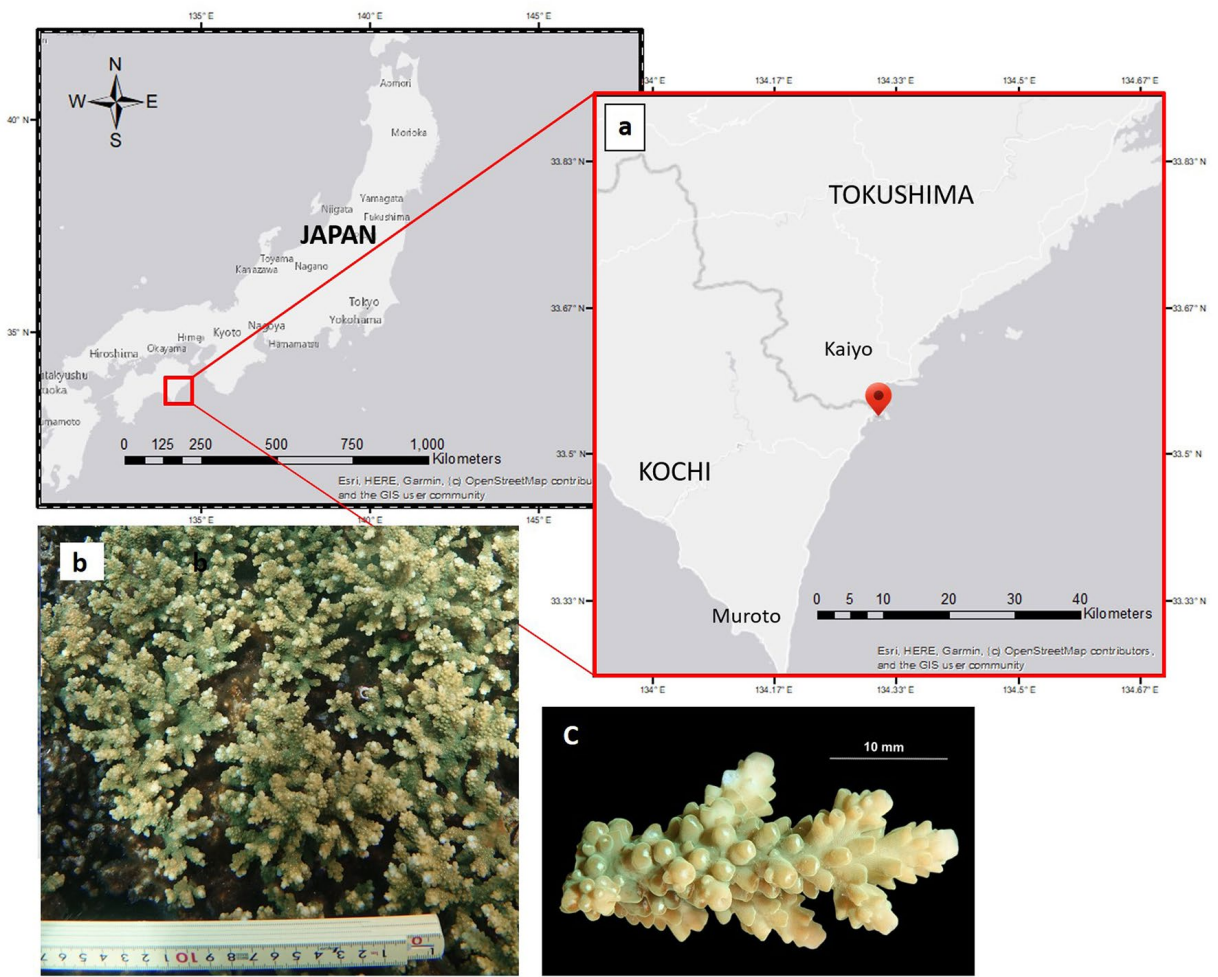
Map showing the location from where *Acropora pruinosa* gametes were obtained and used for artificial fertilization (**a**). The coral colony which released the egg-sperm bundles (**b**). A branch from the colony (**c**). Maps were generated using ArcMap 10.3 (https://desktop.arcgis.com/en/arcmap/).

Embryos of *A. pruinosa* were obtained from artificial fertilization of egg-sperm bundles collected from spawning colonies in Kaiyo-Cho, Tokushima, Japan (33.545°N, 134.315°E) (Fig. 6a) on the night of July 20, 2019. The coral is characterized by indeterminate colony outline (Fig. 6b), with appressed and tubular radial corallites (Fig. 6c)^51^. Embryos were grown in 0.2 µm filtered seawater for 10–14 h and treated with 0.01% (v/v) colchicine followed by the addition of hypotonic solution (seawater: dH_2_0 = 1:1). Other coral embryos used in this study were preserved ones such as *Acropora* muri*cata* collected also in Kaiyo-Cho and another *Acropora pruinosa* collected in Otsuki, Kochi, Japan (32.777°N, 132.731°E). To distinguish *A. pruinosa* collected in Otsuki, Kochi, Japan, the name *A. pruinosa* Kochi was used throughout this study. Chromosomes were prepared from the embryos based on the method described by Taguchi et al.^8^, with slight modifications. About 30–50 embryos were collected by centrifugation and 0.5 mL of Carnoy’s fixative (absolute methanol:glacial acetic acid = 3:1) was added. Lipids were removed by soaking the embryos in diethyl ether for 4–6 h. Cells were centrifuged at 2000 × *g* for 2 min and then resuspended in 0.5 mL of Carnoy’s fixative. A drop of cell suspension was placed on a slide and then flame-dried.

For G-banding, slides were treated with 0.025% trypsin solution for 1 min, and then stained with Giemsa solution diluted with 5% 0.06 M phosphate buffer (pH 6.8). To examine the chromosomal distribution of constitutive heterochromatin, C-banding was performed using the standard barium hydroxide/saline/Giemsa method^52^ with slight modifications. Chromosome slides were treated with 0.2 N HCl at 25 °C for 30 min and then with 5% Ba(OH)_2_ at 50 °C for 1 min. The slides were then soaked in 2X SSC at 60 °C for 30 min. Experimental research, including the collection of the coral bundles, complied with the relevant institutional, national, and international guidelines and legislation.

### PCR and DNA cloning

*A. pruinosa* genomic DNA was extracted from sperms using the Wizard Genomic DNA Purification kit (Promega, USA). The 5S rRNA genes were amplified using the forward primer described by Stover & Steel^53^ and the reverse primer (R: 5′-GGGCCAGGGTAGTACTTGGA-3′) designed by us. Histone genes were amplified using the primers (F: 5′-TTGCAAGTTCACCGGGAAGC-3′, R: 5′-TTCCAGCCAACTCGAGAATC-3′) designed by us based on the partial histone gene sequences of *Acropora* species retrieved from the GenBank. The PCR conditions for all amplifications were as follows: 30 cycles of 98 °C for 20 s, 60 °C for 30 s, and 72 °C for 1 min 30 s.Gel electrophoresis showed the expected size for both genes (Supplementary Figure S1). The PCR products were ligated into a bacterial plasmid using the pGEM-T Easy Vector Systems (Promega, USA) and transformed into JM109 competent cells (Promega, USA). The cells were then spread plated onto Luria broth (LB) plates containing 100 mg/mL of ampicillin, 40 mg/mL of 5-bromo-4-chloro-3-indolyl-β-D-galactoside (X-Gal), and 0.05 mmol/L isopropyl-β-D-thio-galacto-pyranoside (IPTG). The plates were incubated for 15 h, and bacterial colonies were screened for positive inserts using colony PCR followed by gel electrophoresis. Positive colonies were grown in LB medium for 15 h and plasmids were extracted thereafter using Mini *Plus* Plasmid DNA (Viogene, USA). The inserts that were positive in FISH screening were sequenced with M13 universal primers using the ABI Prism BigDye Terminator Cycle Sequencing Ready Reaction Kit ver.2.0 (PE Biosystems, Japan). Primer walking was conducted for insert sizes greater than 1 kbp. The sequence reads were checked, assembled, and vector sequences were removed manually using MEGA X^54^. DNA sequences were submitted to the DNA Data Bank of Japan (DDBJ) with accession numbers LC557012-LC557016.

### Probe preparation and FISH

FISH probes were prepared from the plasmid DNA using the Random Primed DNA Labeling Kit (Roche, USA) according to the manufacturer’s protocol. The DNA was fluorescently labeled directly using cyanine-3-dUTP (Cy3-dUTP) (PerkinElmer, USA) or indirectly using digoxigenin-dUTP (DIG-dUTP)/anti-Digoxigenin-FITC (Roche, USA) at 37 °C for 15–18 h. The probe obtained using 5S rDNA sequence as the target was named At-p5S, whereas that obtained from histone was named At-pH2AB. FISH was performed according to the method described by Taguchi et al^9^, with slight modifications. Slides of *A. pruinosa* chromosomes were denatured in 70% formamide solution at 70 °C for 2 min and then serially submerged in ice-cold 70%, 90%, and 99% EtOH for a total of 6 min. About 1 µL of DNA probes were mixed with 10 µL hybridization solution (H7782, Sigma, Japan) and then denatured at 80 °C for 1 h. For whole genome hybridization experiment, probes were then incubated at 37 °C for 1 h to allow pre-annealing of simple tandem repeats (i.e. G-C repeats). This is to minimize the hybridization signals and reveal clusters composed of high-complexity sequences. The slides with denatured chromosomes were incubated with the probe solution at 37 °C for 12–15 h to allow hybridization. Post hybridization washing was performed with 50% formamide at 43 °C for 20 min and subsequently with 2X SSC at 37 °C for 8 min. The slides were incubated twice in 1X phosphate-buffered detergent (PBD) at 25 °C for 5 min. The chromosomes were then counterstained with DAPI-Vectashield (Vector Laboratories, USA) and viewed under an AxioImager A2 fluorescence microscope equipped with an Axiocam MRm CCD camera (Zeiss, Germany). Images of suitable metaphase spreads from different embryos were captured using the AxioVision software (Zeiss). The FISH images were analyzed by measuring the chromosome lengths and hybridization signal locations using the DRAWID software^55^. Centromere indices (long arm/total length) was computed based on the formula of Lucas and Gray^56^.

## Supplementary Information

Supplementary Information.
