# Publisher Correction: Cytogenetic markers using single-sequence probes reveal chromosomal locations of tandemly repetitive genes in scleractinian coral *Acropora pruinosa*

**DOI:** 10.1038/s41598-021-96115-y

**Published:** 2021-08-12

**Authors:** Joshua Vacarizas, Takahiro Taguchi, Takuma Mezaki, Masatoshi Okumura, Rei Kawakami, Masumi Ito, Satoshi Kubota

**Affiliations:** 1 Kuroshio Science Program, Graduate School of Integrated Arts and Sciences, Kochi University, Nankoku, Kochi Japan; 2 Department of Nutrition, Faculty of Health Sciences, Kochi Gakuen University, Kochi, Kochi Japan; 3 Kuroshio Biological Research Foundation, Otsuki, Kochi Japan; 4 Sea Nature Museum Marine Jam, Kaiyo, Tokushima Japan; 5 Faculty of Agriculture and Marine Science, Kochi University, Nankoku, Kochi Japan

Correction to: *Scientific Reports* 10.1038/s41598-021-90580-1, published online 31 May 2021

The original version of this Article contained an error in Affiliation 4, which was incorrectly given as ‘Sea Nature Museum Marine Jam, Kaiyo, Kochi, Japan’. The correct affiliation is listed below:

Sea Nature Museum Marine Jam, Kaiyo, Tokushima, Japan.

The original Article has been corrected.

