# Supplementary material for: Cytogenetic markers using single-sequence probes reveal chromosomal locations of tandemly repetitive genes in scleractinian coral Acropora pruinosa
Source: Sci Rep. 2021 May 31;11:11326. doi: 10.1038/s41598-021-90580-1 (PMC8167085; doi:10.1038/s41598-021-90580-1)
Supplement: Supplementary file 1 — Supplementary Information. [file 41598_2021_90580_MOESM1_ESM.docx]

Supplementary Table S1. Multiple comparison analysis (Tukey’s test, alpha=0.05) of size differences between homologs of each chromosome pairs. Initial ANOVA analysis (alpha=0.05) is significant (df=13, F=6.4141, p<1.467e-10).

| **contrast** | **estimate** | **SE** | **df** | **t.ratio** | **p.value** |
| --- | --- | --- | --- | --- | --- |
| 1vs2 | 9.45E-02 | 0.0226 | 266 | 4.179 | 0.0031 |
| 1vs3 | 1.53E-01 | 0.0226 | 266 | 6.79 | <.0001 |
| 1vs4 | 1.32E-01 | 0.0226 | 266 | 5.846 | <.0001 |
| 1vs5 | 1.36E-01 | 0.0226 | 266 | 6.035 | <.0001 |
| 1vs6 | 1.46E-01 | 0.0226 | 266 | 6.47 | <.0001 |
| 1vs7 | 1.41E-01 | 0.0226 | 266 | 6.216 | <.0001 |
| 1vs8 | 1.49E-01 | 0.0226 | 266 | 6.598 | <.0001 |
| 1vs9 | 1.57E-01 | 0.0226 | 266 | 6.967 | <.0001 |
| 1vs10 | 1.46E-01 | 0.0226 | 266 | 6.467 | <.0001 |
| 1vs11 | 1.49E-01 | 0.0226 | 266 | 6.591 | <.0001 |
| 1vs12 | 1.36E-01 | 0.0226 | 266 | 6.033 | <.0001 |
| 1vs13 | 1.14E-01 | 0.0226 | 266 | 5.063 | 0.0001 |
| 1vs14 | 1.12E-01 | 0.0226 | 266 | 4.936 | 0.0001 |
| 2vs3 | 5.90E-02 | 0.0226 | 266 | 2.611 | 0.3386 |
| 2vs4 | 3.77E-02 | 0.0226 | 266 | 1.667 | 0.9256 |
| 2vs5 | 4.20E-02 | 0.0226 | 266 | 1.856 | 0.8479 |
| 2vs6 | 5.18E-02 | 0.0226 | 266 | 2.291 | 0.5633 |
| 2vs7 | 4.60E-02 | 0.0226 | 266 | 2.037 | 0.7428 |
| 2vs8 | 5.47E-02 | 0.0226 | 266 | 2.419 | 0.4692 |
| 2vs9 | 6.30E-02 | 0.0226 | 266 | 2.788 | 0.2371 |
| 2vs10 | 5.17E-02 | 0.0226 | 266 | 2.288 | 0.5653 |
| 2vs11 | 5.45E-02 | 0.0226 | 266 | 2.412 | 0.4746 |
| 2vs12 | 4.19E-02 | 0.0226 | 266 | 1.854 | 0.8488 |
| 2vs13 | 2.00E-02 | 0.0226 | 266 | 0.884 | 0.9998 |
| 2vs14 | 1.71E-02 | 0.0226 | 266 | 0.757 | 1 |
| 3vs4 | -2.13E-02 | 0.0226 | 266 | -0.944 | 0.9996 |
| 3vs5 | -1.71E-02 | 0.0226 | 266 | -0.755 | 1 |
| 3vs6 | -7.24E-03 | 0.0226 | 266 | -0.32 | 1 |
| 3vs7 | -1.30E-02 | 0.0226 | 266 | -0.574 | 1 |
| 3vs8 | -4.33E-03 | 0.0226 | 266 | -0.192 | 1 |
| 3vs9 | 4.00E-03 | 0.0226 | 266 | 0.177 | 1 |
| 3vs10 | -7.30E-03 | 0.0226 | 266 | -0.323 | 1 |
| 3vs11 | -4.50E-03 | 0.0226 | 266 | -0.199 | 1 |
| 3vs12 | -1.71E-02 | 0.0226 | 266 | -0.757 | 1 |
| 3vs13 | -3.90E-02 | 0.0226 | 266 | -1.727 | 0.9045 |
| 3vs14 | -4.19E-02 | 0.0226 | 266 | -1.854 | 0.849 |
| 4vs5 | 4.28E-03 | 0.0226 | 266 | 0.189 | 1 |
| 4vs6 | 1.41E-02 | 0.0226 | 266 | 0.624 | 1 |
| 4vs7 | 8.37E-03 | 0.0226 | 266 | 0.37 | 1 |
| 4vs8 | 1.70E-02 | 0.0226 | 266 | 0.752 | 1 |
| 4vs9 | 2.53E-02 | 0.0226 | 266 | 1.121 | 0.9977 |
| 4vs10 | 1.40E-02 | 0.0226 | 266 | 0.621 | 1 |
| 4vs11 | 1.68E-02 | 0.0226 | 266 | 0.745 | 1 |
| 4vs12 | 4.24E-03 | 0.0226 | 266 | 0.187 | 1 |
| 4vs13 | -1.77E-02 | 0.0226 | 266 | -0.783 | 1 |
| 4vs14 | -2.06E-02 | 0.0226 | 266 | -0.91 | 0.9997 |
| 5vs6 | 9.83E-03 | 0.0226 | 266 | 0.435 | 1 |
| 5vs7 | 4.09E-03 | 0.0226 | 266 | 0.181 | 1 |
| 5vs8 | 1.27E-02 | 0.0226 | 266 | 0.563 | 1 |
| 5vs9 | 2.11E-02 | 0.0226 | 266 | 0.932 | 0.9997 |
| 5vs10 | 9.77E-03 | 0.0226 | 266 | 0.432 | 1 |
| 5vs11 | 1.26E-02 | 0.0226 | 266 | 0.556 | 1 |
| 5vs12 | -4.29E-05 | 0.0226 | 266 | -0.002 | 1 |
| 5vs13 | -2.20E-02 | 0.0226 | 266 | -0.972 | 0.9995 |
| 5vs14 | -2.48E-02 | 0.0226 | 266 | -1.099 | 0.9981 |
| 6vs7 | -5.73E-03 | 0.0226 | 266 | -0.254 | 1 |
| 6vs8 | 2.90E-03 | 0.0226 | 266 | 0.128 | 1 |
| 6vs9 | 1.12E-02 | 0.0226 | 266 | 0.497 | 1 |
| 6vs10 | -5.97E-05 | 0.0226 | 266 | -0.003 | 1 |
| 6vs11 | 2.73E-03 | 0.0226 | 266 | 0.121 | 1 |
| 6vs12 | -9.87E-03 | 0.0226 | 266 | -0.437 | 1 |
| 6vs13 | -3.18E-02 | 0.0226 | 266 | -1.407 | 0.9805 |
| 6vs14 | -3.47E-02 | 0.0226 | 266 | -1.534 | 0.9603 |
| 7vs8 | 8.64E-03 | 0.0226 | 266 | 0.382 | 1 |
| 7vs9 | 1.70E-02 | 0.0226 | 266 | 0.751 | 1 |
| 7vs10 | 5.67E-03 | 0.0226 | 266 | 0.251 | 1 |
| 7vs11 | 8.47E-03 | 0.0226 | 266 | 0.375 | 1 |
| 7vs12 | -4.14E-03 | 0.0226 | 266 | -0.183 | 1 |
| 7vs13 | -2.61E-02 | 0.0226 | 266 | -1.153 | 0.9969 |
| 7vs14 | -2.89E-02 | 0.0226 | 266 | -1.28 | 0.9916 |
| 8vs9 | 8.34E-03 | 0.0226 | 266 | 0.369 | 1 |
| 8vs10 | -2.96E-03 | 0.0226 | 266 | -0.131 | 1 |
| 8vs11 | -1.71E-04 | 0.0226 | 266 | -0.008 | 1 |
| 8vs12 | -1.28E-02 | 0.0226 | 266 | -0.565 | 1 |
| 8vs13 | -3.47E-02 | 0.0226 | 266 | -1.536 | 0.9599 |
| 8vs14 | -3.76E-02 | 0.0226 | 266 | -1.662 | 0.9271 |
| 9vs10 | -1.13E-02 | 0.0226 | 266 | -0.5 | 1 |
| 9vs11 | -8.51E-03 | 0.0226 | 266 | -0.376 | 1 |
| 9vs12 | -2.11E-02 | 0.0226 | 266 | -0.934 | 0.9997 |
| 9vs13 | -4.30E-02 | 0.0226 | 266 | -1.904 | 0.8225 |
| 9vs14 | -4.59E-02 | 0.0226 | 266 | -2.031 | 0.7469 |
| 10vs11 | 2.79E-03 | 0.0226 | 266 | 0.124 | 1 |
| 10vs12 | -9.81E-03 | 0.0226 | 266 | -0.434 | 1 |
| 10vs13 | -3.17E-02 | 0.0226 | 266 | -1.404 | 0.9808 |
| 10vs14 | -3.46E-02 | 0.0226 | 266 | -1.531 | 0.9608 |
| 11vs12 | -1.26E-02 | 0.0226 | 266 | -0.558 | 1 |
| 11vs13 | -3.45E-02 | 0.0226 | 266 | -1.528 | 0.9614 |
| 11vs14 | -3.74E-02 | 0.0226 | 266 | -1.654 | 0.9294 |
| 12vs13 | -2.19E-02 | 0.0226 | 266 | -0.97 | 0.9995 |
| 12vs14 | -2.48E-02 | 0.0226 | 266 | -1.097 | 0.9981 |
| 13vs14 | -2.86E-03 | 0.0226 | 266 | -0.126 | 1 |

Supplementary Table S2. Blast result of each identified regions of At-p5S probe sequence against the whole genome of *Acropora digitifera* (GenBank: GCA_014634065.1). Highlighted in yellow is the probe array 5S-ITS1-U2-ITS2-U1-ITS3-5S which aligned on pos 1203043-1626684.

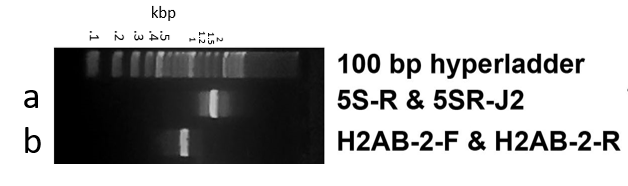


Supplementary Figure S1. Gel electrophoresis image of PCR-amplified repetitive 5S-U1-U2 snRNA (a) and core histone gene (b).
